# Computational framework for the prediction of transcription factor binding sites by multiple data integration

**DOI:** 10.1186/1471-2202-7-S1-S8

**Published:** 2006-10-30

**Authors:** Alberto Ambesi-Impiombato, Mukesh Bansal, Pietro Liò, Diego di Bernardo

**Affiliations:** 1 TIGEM, Telethon Institute of Genetics and Medicine, Naples, Italy; 2 Department of Neuroscience, University of Medicine "Federico II", Naples, Italy; 3 SEMM, European School of Molecular Medicine, Naples, Italy; 4 Computer Laboratory, Cambridge University, Cambridge, UK

## Abstract

Control of gene expression is essential to the establishment and maintenance of all cell types, and its dysregulation is involved in pathogenesis of several diseases. Accurate computational predictions of transcription factor regulation may thus help in understanding complex diseases, including mental disorders in which dysregulation of neural gene expression is thought to play a key role. However, biological mechanisms underlying the regulation of gene expression are not completely understood, and predictions via bioinformatics tools are typically poorly specific.

We developed a bioinformatics workflow for the prediction of transcription factor binding sites from several independent datasets. We show the advantages of integrating information based on evolutionary conservation and gene expression, when tackling the problem of binding site prediction. Consistent results were obtained on a large simulated dataset consisting of 13050 *in silico *promoter sequences, on a set of 161 human gene promoters for which binding sites are known, and on a smaller set of promoters of Myc target genes.

Our computational framework for binding site prediction can integrate multiple sources of data, and its performance was tested on different datasets. Our results show that integrating information from multiple data sources, such as genomic sequence of genes' promoters, conservation over multiple species, and gene expression data, indeed improves the accuracy of computational predictions.

## Background

Control of gene expression is essential to the establishment and maintenance of all cell types, and is involved in pathogenesis of several diseases, possibly including many complex diseases, such as mental disorders [1]. Neuronal gene expression regulation is expected to be more complex than other cell types. It is largely orchestrated by transcription factors (TFs) that activate and repress specific cohorts of genes in both neural and non-neural cells, required for differentiation of adult neural stem cells and is implicated in several neuropathologies including Huntington's disease, epilepsy and ischemia. Possibly all mental disorders including schizophrenia and mood disorders, for which a biological component is strongly supported by evidence, may be caused by a dysregulation of neural gene expression during development or adulthood, rather than by structural variations in proteins [1]. The identification of genes that encode novel targets of neural-specific transcription factor will provide insights into the pathogenesis of mental disorders and in the identification of clinically relevant drug-induced gene expression patterns. Although the possibility of predicting the regulation of gene expression is appealing, the underlying biological mechanisms are not completely understood, and the development of bioinformatics tools capable of accurate predictions is far from trivial. It is known that the mechanisms of regulation of gene expression involve the binding of TFs to regulatory elements on gene promoters, known as Transcription Factor Binding Sites (TFBSs), but attempts to computationally predict such elements in DNA sequences of gene promoters typically yield an excess of false positives.

Computational identification of cis-Regulatory Elements (CREs) is currently based mainly on three different approaches: (i) identification of conserved motifs using interspecies sequence global alignments [2]; (ii) motif-finding algorithms that identify previously unknown motifs that are overrepresented in the promoters of co-expressed genes [3-9]; (iii) computational detection of previously known motifs in promoters of genes for which regulating TFs are unknown [10]. Limitations of the first approach are caused by the high mutation, deletion and insertion rates in gene promoter regions [11] that prevent a correct alignment of the promoter region, and several other reasons, including rearrangements of binding sites within the non-coding regions or changes in regulation of the ortholog genes. The second approach requires a large number of sequences containing a highly overrepresented motif. The third approach seems promising since the quality of the motif models of each TF is increasing, allowing for more accurate predictions of unknown target genes.

Accurate predictions require the use of an appropriate statistical background model of DNA sequence and integration of several sources of data, such as genomic sequence of gene promoters, as well as genomic sequence of ortholog genes, and gene expression data. Different strategies have been proposed to improve the accuracy of predictions, such as using a statistical background model or the information vector of a position weight matrix (PWM) [10], or, more recently, motif co-occurrence [12]. A promising approach was recently shown to successfully predict TFBSs in higher eukaryotic genomes by considering overrepresented combinations of motifs in phylogenetically conserved regions and correlate them with expression profiles [13].

Tadesse *et al*. [14] could successfully improve specificity of the identification of DNA regulatory motifs by fitting a linear regression model to microarray data in yeast. A novel computational tool was recently released by Hallikas *et al*. [15] for the prediction of distal enhancer elements in mammalian genomes, based on both genomic sequence and conservation. This method tries to detect highly conserved sequences containing clusters of TFBSs by aligning large stretches (50 kb) of genomic DNA from two species. Our focus is somewhat complimentary, as we try to detect TFBSs in the proximal promoter of vertebrate genes as opposed to distal enhancers. Proximal promoters cannot be easily aligned with promoters of ortholog genes, however, our method takes conservation into account in a way that does not require alignment. Conlon *et al*. [16] showed recently that integration of gene expression profiles and PWM scores through a linear regression analysis can indeed improve the prediction accuracy.

**Figure 1:**
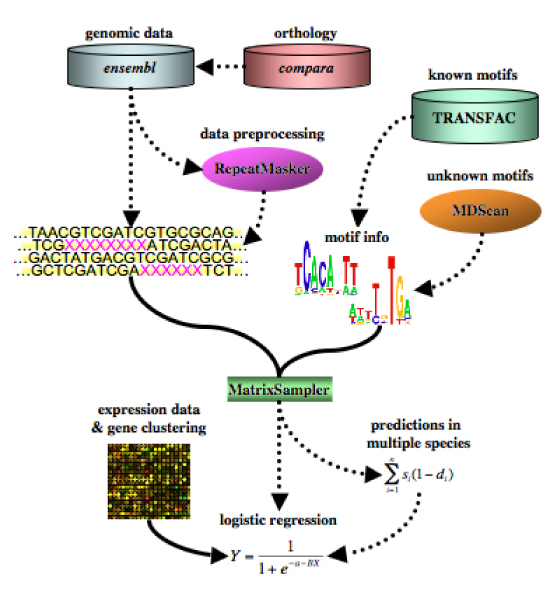
**CFBI Overview**. Diagram illustrating the structure of the framework for the computational prediction of transcription factor binding sites. The diagram shows the multiple sources of input data, including *ensembl*, *compara*, TRANSFAC (or alternatively, novel motifs obtained by a motif-finding algorithm, such as MDScan), the optional data preprocessing RepeatMasker step, and the post-processing steps including data integration from multiple species and logistic regression of gene expression defined classes of genes. Dotted lines indicate optional or alternative steps.

Our Computational Framework for transcription factor Binding site Identification (CFBI) supplies a set of novel tools to fetch and integrate data from multiple sources and analyze it to make predictions, all in an automated and flexible bioinformatics workflow (Figure 1). Differently from previous approaches, CFBI does not require alignment of ortholog gene promoters, nor a linearity assumption, as in the case of linear regression based algorithms. Our framework can also be applied to qualitative expression data, such as developmental and/or neuroanatomical expression data such as that obtained by *in situ *hybridization histochemistry.

## Results

The CFBI approach we developed proceeds as follows (Figure 1): the gene of interest is selected and its promoter sequence, together with promoter sequences of ortholog genes in other species are retrieved from *ensembl *database  and *compara *for orthology information [17]. A list of motifs of all known vertebrate transcription factors (TFs) is obtained by the TRANSFAC database, or a list of novel motifs may be predicted by MDScan [18]. Motifs are then modeled as Position Weight Matrices (PWMs). A PWM score for each motif is computed in each promoter of the ortholog gene set. The PWM scores in the ortholog gene set are integrated using a weighted sum calibrated on the phylogenetic distances between the species. This final score can then be used to rank the motifs and select the ones with the highest probability of being functional transcription factor binding sites.

These predictions can be refined using logistic regression to integrate data from potentially co-regulated genes. The logistic regression makes use of two sets: a set of promoters of potentially co-regulated genes, and a background set of gene promoters that do share any regulatory motifs. For further details please refer to the Methods section.

In order to establish the performance of CFBI, we counted the number of true positives (TP), true negatives (TN), false positives (FP), false negatives (FN), and presented the results as Positive Predictive Value (PPV) = , and Sensitivity = .

### Simulated data

Performance and usability of the CFBI was tested on an *in silico *dataset consisting of 1450 genes with ortholog sequences in 9 different species (see Methods).

**Figure 2:**
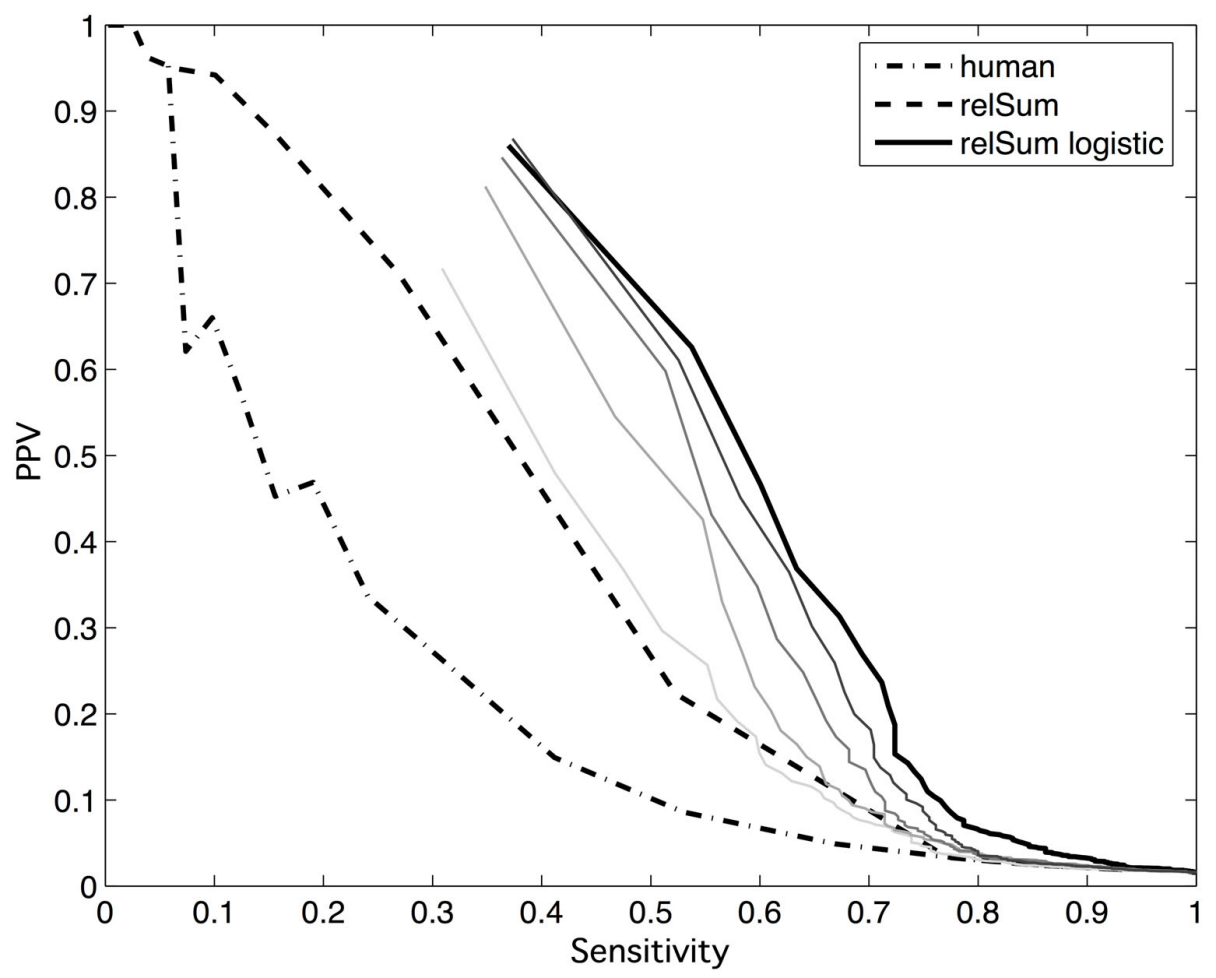
**Performance on the Simulated Dataset**. Positive Predictive Value (PPV) *vs*. Sensitivity plot showing the results in the simulated dataset. Continuous lines: performance profile obtained using the logistic regression step (black thick line shows performance with zero noise, and thin gray scale lines show performance when miss-assignmets are progressively introduced).

The predictive performance of CFBI on this dataset is shown in Figure 2. Robustness of the logistic regression step was tested by progressively introducing 'noise' in the set of co-regulated genes and in the background set of genes (see Methods). Noise was added to simulate a more realistic scenario, in which only some of the genes in the co-regulated set, do share a common regulatory motif in their promoters. The noise free case (black continuous line in Figure 2) consisted of the 10 motif-positive promoters assigned to the co-regulated set of genes, and the null promoters (with no insertions) assigned to the background set. Promoters in the background set were progressively misassigned to the co-regulated set, and the corresponding performances are shown in Figure 2.

### TRANSFAC genes dataset

The TRANSFAC dataset consists of promoters of 407 human genes from TRANSFAC gene table, for which transcription factors are known and experimentally validated with an annotated 5'-UTR. Ortholog gene sequences were fetched via the automated workflow, for each of 9 species where available. The analysis was limited to the subset of 161 groups of ortholog genes for which all 9 orthologs were available, for a total of 1449 promoter sequences. All promoters were 1 kb long, with 300 bp downstream of the transcript start site.

**Figure 3:**
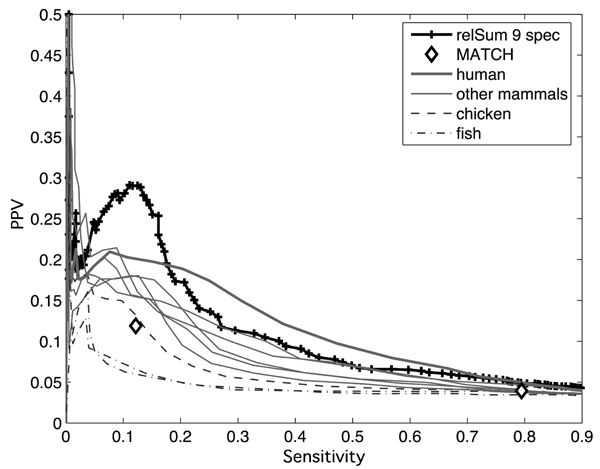
**Performance on the TRANSFAC Genes Dataset**. PPV *vs*. Sensitivity on the TRANSFAC genes dataset. Plain gray lines: scores obtained on the individual species; continuous lines: mammals (the thicker line is the human); dashed lines: chicken; dot dashed: fugu and zebrafish. Performance obtained using MATCH: two bordered white diamonds correspond to 'minimize false positives' and 'minimize false negatives'.

Results on the human TRANSFAC genes dataset confirm the results obtained on the simulated dataset. Single species performance appears to resemble the evolutionary distance of the species (Figure 3). The PPV reached a maximum of approximately 30% when the ortholog gene promoter sequences are used, as compared to an average peak of <20% for the human species alone. We also compared the performance of CFBI with one of the most commonly used algorithms for TFBS prediction, MATCH [10] using both the 'minimize FP' and 'minimize FN' options (Figure 3).

### Myc targets dataset

In order to confirm our results on an independent dataset, we selected a subset of Myc target genes from the Myc database [19]. The Myc gene a transcription factor vastly implicated in neuroscience [20-22], whose primary targets have been extensively validated. Only the top 17 high quality targets were included in the analysis, *i.e*. those validated as primary targets by both Chromatin ImmunoPrecipitation (ChIP) and biochemical assays, in order to have a small but highly reliable dataset [19].

**Figure 4:**
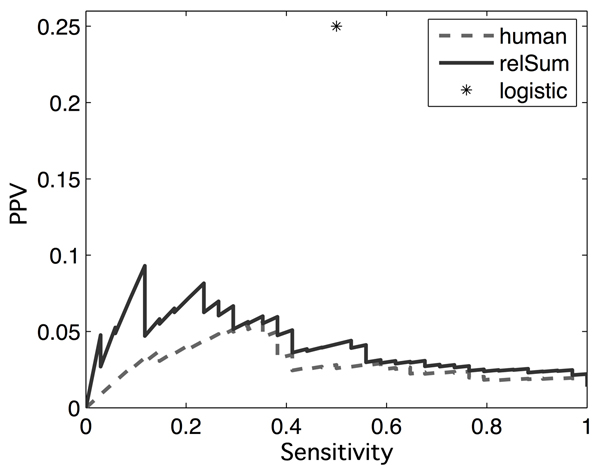
**Performance on the Myc Targets Dataset**. PPV *vs*. Sensitivity on the small set of 'high quality' Myc target genes dataset. Continuous line: performance of the weighted sum over 9 species; dashed line: human alone. The asterisk shows the peak performance obtained by the logistic of the 17 *relSum *scores against 100 promoters of random genes not included in the Myc database.

Performance on this dataset confirms the advantage of integrating phylogenetic sequence information over using a single species, and a boost (> two fold) in performance when integrating information on co-regulated genes via logistic regression (Figure 4).

## Discussion and Conclusion

Regulation of gene expression is a key factor determining complexity of biological systems. There is an increasing interest in understanding regulation of gene expression in the brain, where the dynamics of gene expression may play a role in drug response and in brain disorders. There are examples in which neural gene expression profiles could accurately discriminate among classes of psychoactive compounds [23,24] or even between complex social behaviors within honeybees [25].

Here, we developed a novel strategy for increasing the accuracy of computational predictions of TFBSs on genomic DNA sequences. Key factors of our computational framework include the integration of phylogenetic information from multiple species, and the possibility to include *a priori *information such as that available from quantitative or qualitative gene expression data.

One novelty of our approach, compared to others that make use of phylogenetic information, is that it does not require aligning promoter sequences from different species, thus overcoming the problem of aligning promoter sequences that have diverged with evolution.

A second novelty is the use of non-linear logistic regression to integrate additional *a priori *information on gene regulation. The source of *a priori *information could be microarray gene expression profiles. Clusters of genes that share a common expression profile with a gene of interest can be identified, and considered against a set of genes that do not change. The hypothesis is that genes that are co-expressed should be co-regulated and therefore share common regulatory motifs in their promoters, while the second set of non-changing genes is used as a background set to reduce false positives. Alternatively, contrasting sets of genes could be identified from biological knowledge or from different experimental data such as a specific pattern of expression by *in situ *hybridization. For example, a pattern of expression in specific neuroanatomical regions in response to a drug may be used to select one group of genes, whereas a (larger) set of genes not responding, or responding with a different pattern may be used as the background set. Logistic regression is different from the linear regression method by Conlon *et al*. [14], in that the linear regression model relies on the assumption that the gene expression levels are linearly related to the sequence matching scores of the motifs. Such an effect could be true in lower animals but is not easy to detect in mammals. In addition, the use a background set makes logistic regression less prone to false positive predictions.

## Methods

### Sequence and motif data retrieval

Promoter sequences were retrieved from the latest build of *ensembl *database (build 32), and ortholog gene IDs were obtained by querying the *compara *database [17]. Finally, all of the 145 vertebrate motif data were fetched from TRANSFAC 9.2. Each transcription binding site motif was modeled as a position weight matrix (PWM).

### *Position weight matrix *score

We computed PWM scores using a statistical formula proposed by Stormo *et al*. [26,27]. This score is based on the ratio between the probability of a subsequence being generated from the PWM over that of being generated by the background Markov model. The score of a motif of length *w *over a promoter sequence of length *l *is given by:



where *p*_*ij *_is the probability of a base at position *i+j *based on the PWM and *p*_*'ij *_ is the probability of it being generated by the background Markov model. For this purpose a species-specific 3^rd ^order Markov model was trained on large (10 kb) intergenic regions upstream of a set of human neural genes, including dopamine D_2 _receptor, 5-HT2A, tryptophan hydroxylase 1, homer 1, neuronal acetylcholine receptor alpha-10, c-myc and c-fos. Alternatively, a different set of background sequences may be specified each time.

### Phylogenetic data integration

For each motif, the PWM score in the promoter of ortholog genes in *k *different species was integrated by the following mathematical formula that is based on the assumption that some of the regulatory machinery of gene expression is conserved in evolutionary related species:



where *s*_*i *_is the PWM score of the motif in the promoter of the gene in species *i*, and *d*_*i *_is a weight proportional to evolutionary distance from the main species (human), ranging from 0 (same species) to 1 (farthest species). The distance weight *d*_*i *_was calculated using the multi-species alignment of coding sequences of the *myc *gene using the program DNADIST [28]. We named this score 'relatedness sum' (*relSum*, for short) since it takes into account how related promoters of different species are.

### Qualitative data integration: Logistic regression

If *a priori *information is available indicating that a gene of interest is part of a set of genes that may share common regulatory motifs in their promoters, then this information can be used to increase the specificity of *in silico *predictions. This *a priori *information can be obtained, for example, by selecting a cluster of co-expressed genes from microarray experiments. A value *y *= 1 is assigned to the cluster of co-expressed genes to which the gene of interest belongs, while a value *y *= 0 is assigned to a background set of genes that are thought not to share any common regulatory motifs. Logistic regression is then used to identify the shared regulatory motifs in the co-expressed dataset. The general model for a logistic regression is:



where *n *is the total number of target genes in the two sets, the response variable *y*_*i*_*ε*{0,1} is equal to the class of the *i*^*th *^gene, and **x **is a vector of scores (*relSum*s) for *m *'candidate' motifs (regressors). The vectors **a **and **b **are the parameters of the model. Parameter **b **is a vector of size *m *of fitted weights. The greater the weight, the more likely the corresponding motif is functional. The variance σ^2 ^of **b **may be computed as:



where X is the *n *x *m *matrix of *relSum *scores and the diagonal matrix *w *is equal to:



### Generation of the *in silico *promoter dataset

The sequence generator *Seq-gen *algorithm [29] was used to build simulated datasets of ortholog sequences. *Seq-gen *is able to generate simulated DNA sequences of a given length and the corresponding 'ortholog' sequences at different evolutionary distances, starting from the 9-species phylogenetic distance matrix previously described (Table 1). *Seq-gen *was run to generate 90 simulated DNA sequences with the corresponding 'ortholog' in 9 species (human, chimp, dog, cow, mouse, rat, chicken, fugu, and zebrafish). This program implemented the Hasegawa, Kishino and Yano (HKY) model [30] for the generation of simulated data. Motif sequences were randomly selected from the list of known binding sites in TRANSFAC, and inserted in random non-overlapping positions within the simulated promoter sequences. In order to account for the evolutionary distance, we decreased the frequencies of inserted motifs with the evolutionary distance. Thus, human and chimp promoters received two inserts, cow and dog received 1.5 inserts on average, mouse and rat 1 insert, chicken 0.5 inserts and finally fugu and zebrafish 0.2 inserts. Only the high quality subset of 145 TRANSFAC matrices, *i.e*. compiled from 20 or more binding sites, was considered for the generation of simulated datasets. Thus a total of 13050 promoters were analyzed (145 different datasets of 90 genes).

## Authors' contributions

AA participated in the project's design, wrote the programming code, and drafted the manuscript. MB participated in the statistical analysis. PL participated in the statistical modeling and study design. DdB participated in the study design, project coordination and statistical analysis. All authors read and approved the final manuscript.

## Figures and Tables

**Table 1:** Phylogenetic Distances. Phylogenetic distance weights used to compute the 'relatedness sum' score (variable *d *in equation 2).

**Species**	**distance**
Human	0
Chimp	0.0041
Dog	0.0954
Cow	0.1071
Mouse	0.2595
Rat	0.1352
Chicken	0.3705
Fugu	0.4805
Zebrafish	0.7485
